# Reach, Usage, and Effectiveness of a Medicaid Patient Navigator Intervention to Increase Colorectal Cancer Screening, Cape Fear, North Carolina, 2011

**DOI:** 10.5888/pcd10.120221

**Published:** 2013-05-23

**Authors:** Lucia A. Leone, Daniel S. Reuland, Carmen L. Lewis, Mary Ingle, Brian Erman, Tyana J. Summers, C. Annette DuBard, Michael P. Pignone

**Affiliations:** Author Affiliations: Daniel S. Reuland, Carmen L. Lewis, Brian Erman, Michael P. Pignone, Lineberger Comprehensive Cancer Center, University of North Carolina at Chapel Hill, Chapel Hill, North Carolina; Mary Ingle, Community Care of Lower Cape Fear, Wilmington North Carolina; Tyana J. Summers, C. Annette DuBard, Community Care of North Carolina, Raleigh North Carolina.

## Abstract

**Introduction:**

Screening for colorectal cancer can reduce incidence and death, but screening is underused, especially among vulnerable groups such as Medicaid patients. Effective interventions are needed to increase screening frequency. Our study consisted of a controlled trial of an intervention designed to improve colorectal cancer screening among Medicaid patients in North Carolina.

**Methods:**

The intervention included a mailed screening reminder letter and decision aid followed by telephone support from an offsite, Medicaid-based, patient navigator. The study included 12 clinical practices, 6 as intervention practices and 6 as matched controls. Eligible patients were aged 50 years or older, covered by Medicaid, and identified from Medicaid claims data as not current with colorectal cancer screening recommendations. We reviewed Medicaid claims data at 6 months and conducted multivariate logistic regression to compare participant screening in intervention practices with participants in control practices. We controlled for sociodemographic characteristics.

**Results:**

Most of the sample was black (53.1%) and female (57.2%); the average age was 56.5 years. On the basis of Medicaid claims, 9.2% of intervention participants (n = 22/240) had had a colorectal cancer screening at the 6-month review, compared with 7.5% of control patients (n = 13/174). The adjusted odds ratio when controlling for age, comorbidities, race, sex, and continuous Medicaid eligibility was 1.44 (95% confidence interval, 0.68–3.06). The patient navigator reached 44 participants (27.6%).

**Conclusion:**

The intervention had limited reach and little effect after 6 months on the number of participants screened. Higher-intensity interventions, such as use of practice-based navigators, may be needed to reach and improve screening rates in vulnerable populations.

## Introduction

Colorectal cancer (CRC) screening is effective at reducing both CRC incidence and mortality (1) but is underused; only 65.4% of age-eligible US adults were current with screening guidelines in 2010 (2). Screening is lower for Medicaid recipients (55.4%) compared with privately insured people (69.8%) both nationally and in North Carolina (3–5).

Effective interventions for increasing CRC screening exist (6), but it is unlikely that any single intervention can address the multiple screening barriers encountered at the patient, provider, clinic, and system levels. For example, mailed screening reminders and decision aids are an effective, low-cost, high-reach approach for increasing intention to obtain CRC screening (7,8), but reminders do not address barriers that arise after a person has decided to get screened. Patient navigation can both address individual-level screening barriers and help patients overcome clinical practice and system-level barriers. Patient navigation may also be particularly useful at increasing screening among vulnerable groups (9–11); however, navigation services can be limited by low percentage of populations reached and high cost. Interventions employing multiple methods that combine high-reach, modest-efficacy interventions (ie, mailed reminders or decision aids) with targeted use of higher-efficacy, higher-cost interventions (ie, patient navigation) may increase screening of vulnerable groups and direct more costly care to patients with significant barriers to screening. Additionally, these interventions may be more effective if they can be implemented by insurers and quality-improvement organizations.

In our study, we tested a combined intervention consisting of a mailed screening reminder and decision aid followed by telephone-based patient navigation. The main study objectives were to 1) determine intervention effectiveness by comparing CRC screening rates among patients in intervention practices with patients in matched control practices, at 6 months and 1 year; 2) measure the reach and use of the intervention components; and 3) estimate intervention efficacy by examining screening rates for intervention participants who used intervention components versus those who did not.

## Methods

Researchers at the University of North Carolina at Chapel Hill (UNC) conducted this study from February through September 2011 in partnership with Community Care of North Carolina (CCNC), a statewide, community-based system of managed care for Medicaid recipients that links recipients to primary care practices. Practices are organized into 14 regional networks for coordination of quality improvement and care management activities. We worked with CCNC to identify regional networks that would be amenable to participation and subsequently chose Community Care of Lower Cape Fear (CCLCF), based in southeastern North Carolina. All study methods were approved by the institutional review board of UNC.

### Eligibility and recruitment of practices and participants

In order to ensure heterogeneity and improve generalizability, clinical and executive leadership from CCLCF selected a set of 6 intervention practices that differed on the following characteristics: size, geographic region (urban vs rural), and whether or not the practices trained medical residents. Our goal was to select practices that together had a total of 250 to 300 unscreened patients aged 50 to 74 years. For the purpose of selecting practices, we determined the number of patients who were not up-to-date for screening by using Medicaid claims as of January 1, 2011. CCLCF’s medical director sent the practices selected for the intervention information about the study and asked for their participation; all practices approached agreed to participate. We conducted meetings with leaders at the participating intervention practices in February and March of 2011 to familiarize them with the study and collect basic information about each practice. CCLCF also identified a set of 6 matched control practices that were similar to intervention practices on the selection criteria (size, location, medical residents) to serve as a comparison group.

The number of primary care physicians at the 6 intervention practices ranged from 1 to 17. The number of non-Medicare eligible Medicaid patients aged 50 to 75 years that these practices served ranged from 25 to 149; the baseline CRC screening rates among the intervention practices ranged from 30.0% to 52.0% (mean, 35.6%). The eligible population at control practices ranged from 27 to 146, and screening rates for the target population in those practices ranged from 25.9% to 52.1% (mean = 46.0%).

Eligible patients were aged 50 to 74 years, currently enrolled in Medicaid, not enrolled in Medicare, and not currently meeting CRC screening recommendations (1). We determined if a patient had been screened by using Medicaid claims data as of the date of the practice meeting for intervention practices; the same date was used for the matched control practices. We requested and received a waiver of informed consent from intervention patients. We did not contact or enroll control patients, but received de-identified screening data compiled by CCNC on the basis of Medicaid claims.

### Intervention

Approximately 2 months after the initial meeting with the clinical intervention practices, the navigator mailed all eligible intervention patients a packet containing a letter from their physician on the physician’s practice letterhead. For 2 practices where policy did not allow the letter to come from the physician, the letter came from the patient navigator on CCLCF letterhead. The letter indicated that the patients needed to be screened for CRC and invited them to participate in the study. The packet contained an information sheet explaining the study, a survey, and an updated version of a previously tested CRC screening decision aid (12). The decision aid, called CHOICE, is an 11-minute DVD that provides information about colorectal cancer and the different tests that screen for this cancer. The DVD included testimonials from people who have been screened and a comparison of colonoscopy and the stool blood test screening method. The decision aid promotes screening and emphasizes that patients can choose the test that is best for them.

The mailing included an option to decline participation in data collection and any further intervention by mail or by telephone. The letter directed those who wished to participate to watch the decision aid DVD before completing and returning the survey. It also gave participants the option to contact the patient navigator directly with any questions or concerns.

CCLCF leadership selected a Medicaid patient outreach coordinator from their staff to serve as the patient navigator. The patient navigator participated in a 2-day training that covered CRC and CRC-screening information, motivational interviewing techniques (13), how to work with patients at different stages of change, and how to address practical barriers to screening (14). We adapted training materials from previous studies (15,16). After the training, the patient navigator conducted several mock telephone calls; the research team monitored the calls and provided feedback.

One month after mailing the packet to intervention participants, the patient navigator made 3 attempts to call each patient who had not opted out of the study. She worked with the patient’s practice staff to resolve disconnected or nonworking telephone numbers. When a patient was reached, the navigator identified herself as a Medicaid patient navigator working with the patient’s physician. She confirmed that the patient had received the information packet and offered the patient the opportunity to complete the survey on the telephone if the patient had not already returned it by mail. The patient navigator then offered her assistance to anyone except those who opted out, were determined ineligible because of recent screening, or were unable to communicate in English. She assessed the participants’ stages of change and used motivational interviewing techniques to encourage screening and make a decision about whether or not to get screening and screening type. She helped participants address barriers, assisted with scheduling and transportation, called to remind participants about appointments, and ensured that patients completed the preparation and testing properly. The patient navigator attempted to follow up with patients until the 6-month intervention period ended or the patient declined further contact.

### Data collection

We used North Carolina Medicaid claims data to identify all Medicaid patients at participating clinical practices who were not up-to-date with CRC screening at baseline. We queried the claims data for both intervention and control patients to identify any CRC screenings that occurred within the 6-month intervention period, and we made identical queries at 1 year. We used Medicaid records to collect data on sex, age, race, and co-morbidities and to determine whether the patient had been enrolled in Medicaid continuously throughout the study period or if they had become eligible for Medicare during that period.

A trained CCNC care manager who was not a member of the study team conducted chart reviews for intervention patients. The chart review looked for evidence of any discussion, ordering, or completion of a CRC screening test during the intervention period, the date of the test if it had been completed, and the dates of all primary care visits during the intervention period.

We included the participant survey in the initial packet mailed to intervention participants; the letter asked interested participants to complete the survey after watching the DVD. Participants who did not return the survey by mail had the option to complete it over the telephone with the navigator. We mailed anyone who completed the survey a $10 gift card. The survey included questions on screening history, screening intentions, patient and family history related to CRC and other risk factors, demographics, and the participant’s opinions about the DVD decision aid.

### Assessing study outcomes

We used Medicaid claims to determine whether or not a participant had completed any CRC screening test during the 6-month intervention period for intervention and control patients who were eligible for the intervention on the basis of claims data only. We also added a secondary outcome of completion of a CRC screening test at 12 months to account for delays in screening test completion and Medicaid claims processing. For this intention-to-treat analysis, we did not exclude anyone from the intervention group so that the sample would be most similar to the matched controls who had no option to opt out or self-report as ineligible.

We examined intervention reach by determining the percentage of intervention participants who received or used the different components of the intervention. We measured decision aid use by using responses to questions on the participant survey. We determined contact with the patient navigator based on the telephone logs the navigator kept.

To estimate the efficacy of individual intervention components, we compared screening odds for participants who received each intervention component with those who did not. For this analysis, we excluded participants who were not eligible for the study because they were up-to-date for screening at baseline (determined by either chart review or Medicaid claims data) or who opted out or self-reported as ineligible. We considered participants to have received a screening during the intervention period if the screening was reported in either the Medicaid claims or chart review data.

### Analyses

We completed all analyses with SAS Version 9.2 (SAS Institute, Inc, Cary, North Carolina) and used statistical procedures to account for patient clustering within practices. We used Rao-Scott χ^2^ to test differences in categorical variables between intervention and control groups and between subgroups. To compare screening between intervention and control patients, we used multivariate logistic regression including fixed-effect terms for experimental condition and to adjust for clustering within practices and potential confounders. For the adjusted analysis, we excluded intervention patients who opted out of the study (n = 27) because covariate data were not available for this group.

## Results

### Participant characteristics

On the basis of the initial review of Medicaid claims, 242 patients at intervention practices were eligible for the study (Figure). Of patients at control practices, 174 were eligible for the study. Of this initial sample, 57.2% were female, and average age was 56.5 years; 98.3% were aged less than 65 years (Table 1). Race differed between groups: 62.0% of intervention participants were black compared with 40.8% of control participants. On the basis of chart reviews, 83.7% of participants had at least 1 visit to the physician practice during the intervention period; the average of number of visits was 1.9 (95% CI: 1.6–2.2).

**Figure:**
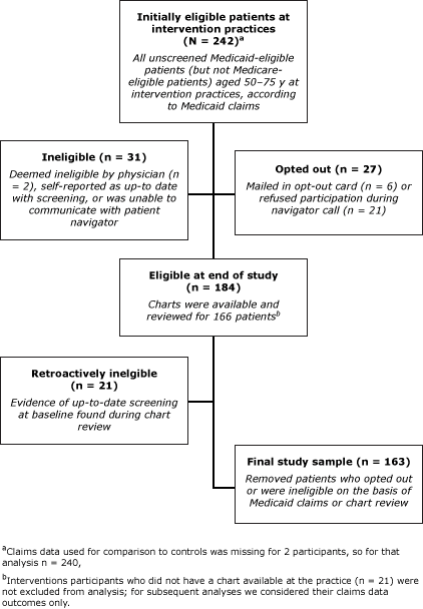
Selection of eligible patients from intervention practices to study the reach, usage, and effectiveness of a Medicaid patient navigator intervention to increase colorectal cancer screening, Cape Fear, North Carolina, 2011. Medicaid claims data used for comparison with controls was missing for 2 participants; for that analysis n = 240.

**Table 1:** Baseline Characteristics of Intervention and Control Patients Using Medicaid Data, Intervention to Increase Colorectal Cancer Screening, North Carolina, 2011^a^

Characteristic	Entire Sample (N = 416), N (%)	Intervention (n = 242), %	Control, %	*P* Value^b^
**Female**	238 (57.2)	57.0	57.5	.09
**Age, y**				
50–54	167 (40.1)	39.7	40.8	.87
55–59	132 (31.7)	31.8	31.6	
60–64	110 (26.4)	26.4	26.4	
≥65	7 (1.7)	2.1	1.1	
**Race**				
Black	221 (53.1)	62.0	40.8	<.001
White	167 (40.1)	31.0	52.9	
Other^c^	4 (1.0)	0.41	1.7	
Unreported	24 (5.8)	6.6	4.6	
**Became eligible for Medicare during study**	19 (4.5)	15.6	2.9	.10
**Had continuous Medicaid eligibility during study**	378 (89.4)	88.8	90.2	.67
**Age, y, mean (SE)**	56.5 (0.38)	56.5 (0.34)	56.2 (0.26)	.52
**Comorbidities score^d^, mean (SE)**	3.9 (0.30)	3.5 (0.22)	4.4 (0.41)	.02

a All data in table are from Medicaid records and are n (%) unless otherwise indicated.

b *P* value comparing intervention versus control participants calculated by using Rao-Scott χ^2^ test adjusted for cluster randomized design (categorical variables) or *t* tests for continuous variables.

c Other races include Native Hawaiian/Pacific Islander, Asian, and American Indian/Alaska Native.

d The comorbidities score is from Medicaid’s Clinical Risk Group weighting system (http://www.ncbi.nlm.nih.gov/pubmed/14713742), which assigns scores to patients on the basis of a prediction of resources needed to address their conditions. For example, a weight of 3 indicates that a patient is expected to spend 3 times the time of the average patient.

### Intervention effectiveness

Medicaid claims data were missing at follow-up for 2 of the initial 242-person sample of intervention participants, leaving 240. Medicaid claims data showed that 9.2% of intervention participants (n = 22), had a CRC screening test during the 6-month intervention period compared with 7.5% of control participants (n = 13; unadjusted odds ratio [OR], 1.25; 95% confidence interval [CI], 0.63–2.50). After controlling for age, comorbidities, race, sex, and continuous Medicaid eligibility, the adjusted OR was 1.44 (95% CI, 0.68–3.06). Claims data were reviewed again at 1 year, and we found that 16.3% of intervention participants had been screened compared with 10.3% of control patients. Screening was still not significantly higher in the intervention group (unadjusted OR, 1.68; 95% CI, 0.80–3.56).

### Intervention reach, use, and efficacy

All of the 242 intervention patients that we originally categorized as unscreened on the basis of Medicaid claims data were offered the intervention, however for the purpose of determining intervention efficacy, we excluded anyone from the analysis who opted out of the study (n = 27), did not meet eligibility criteria (n = 31), or whose chart review indicated previous CRC screening (n = 21), resulting in 163 eligible intervention participants (Figure). The overall screening rate within this group considering both claims and chart review data was 12.9% (21/163) (Table 2).

**Table 2:** Colorectal Cancer Screening Outcomes at 6 Months for Intervention Participants According to Intervention Exposure, Intervention to Increase Colorectal Cancer Screening, North Carolina, 2011^a^

Process Variable	Reach	Any Screening During Study^b^	*P* Value^c^
	**n (%)**	**% (95% Confidence Interval^c^)**	
**Eligible intervention participants**	163 (100)	12.9 (NA)	(NA)
**Completed the survey**			
Yes	56 (34.4)	23.2 (16.6–29.8)	.004
No	107 (65.6)	7.5 (0-15.8)	
**Watched decision aid (survey completers only^d^)**			
Some or all	21 (50.0)	28.6 (10.4–46.7)	.31
No	21 (50.0)	19.0 (3.0–35.0)	
**Spoke with patient navigator**			
Yes	45 (27.6)	22.2 (11.7–32.7)	<.001
No	118 (72.4)	9.3 (3.8–14.8)	
**Had follow-up call (among those who spoke with patient navigator)**			
Yes	26 (57.8)	30.8 (12.1–49.4)	.12
No	19 (42.2)	6.6 (0-27.6)	
**Had physician visit during study period (among patients with chart reviews only^e^)**			
Yes	118 (84.3)	14.4 (8.1–20.7)	.10
No	23 (16.3)	4.3 (0.0–12.0)	

Abbreviation: NA, not applicable.

a Table includes only participants who did not refuse and were not up-to-date for screening at baseline on the basis of self-report, Medicaid claims data, or chart review data (n = 163). For this analysis we looked at only the 163 (out of the initial 242) intervention participants who met all eligibility criteria based on both chart review and Medicaid claims data. We excluded 27 people who opted out of the study, 31 people who self-reported as ineligible, and 21 people who were determined to be retroactively ineligible because their chart review indicated that they had had been up-to-date with CRC screening at baseline.

b Includes evidence or screening during the intervention period found in chart reviews or Medicaid claims data.

c *P* values and 95% confidence intervals are calculated by using χ^2^ test and account for clustering by practice

d Excludes people who answered the survey, but skipped the decision aid questions (n = 14).

e Excludes 2 patients for whom data on visits was missing on their chart review (n=141)

Among the 163 intervention participants with complete data from both sources, 56 people (34.4%) completed the survey (Table 2). Participants who completed the survey had greater odds of being screened at follow-up (OR, 4.4, 95% CI: 1.4–13.9). The survey asked participants if they had watched the decision aid; 42 of 56 participants who completed the survey responded to this question. Among those who answered the question, 50% indicated that they had watched some or all of the decision aid. The odds of getting screened was higher for those reported watching some or all of the decision aid compared with those who did not watch it (OR, 1.4; 95% CI, 0.4–5.7), but these differences were not significant.

The patient navigator was able to reach 44 (27.6%) participants. Over half of 242 intervention patients that we attempted to contact were not reached because of wrong or disconnected numbers (n = 59) or because they failed to answer after 3 telephone call attempts (n = 69). The odds of getting screened were higher for those who were reached versus those who were not (OR, 3.5; 95% CI, 1.7–7.1). Among patients with chart review data (n=142), we found that only 16.2% (n = 23) of patients had no reported provider visits during the intervention period. The odds of getting screened were not significantly higher among patients who had a visit during the study period compared with those who did not (OR, 3.5; 95% CI, 0.8–15.0).

### Comparison of charts and claims data

Medicaid claims data indicated that 8.7% (n = 16) of eligible intervention patients who did not refuse participation (n = 184) were screened during the 6-month intervention period (Table 3). For the chart-review only analysis, we looked at participants (n=142/184) who were unscreened at baseline based on their chart reviews; we excluded anyone for whom chart review data was unavailable (n = 21) or who was up-to-date with CRC screening at baseline based on the chart review (n = 21). Similarly, data indicated that 8.5% (12/142) had been screened on the basis of chart review data only. Combining the data from both sources yielded a higher screening rate of 12.9% (21/163). Among those who had a visit, but were not screened during the intervention period (n = 130), 30 additional patient charts showed that CRC screening had either been discussed or ordered (23.1%).

**Table 3:** Colorectal Cancer Screening Outcomes at 6 Months for Intervention Participants Based on Chart or Medicaid Claims Data, Intervention to Increase Colorectal Cancer Screening, North Carolina, 2011^a^

Colorectal Cancer Screening	Medicaid Claims Only (n = 184), N (%)	Chart Review Only^b^ (n = 142), N (%)	Combined Medicaid Claims and Chart Review Data^c^ (n = 163), N (%)
Any screening during study	16 (8.7)	12 (8.5)	21 (12.9)
Colonoscopy during study	11 (6.0)	9 (6.3)	14 (8.6)
Fecal occult blood test during study	13 (7.1)	3 (2.1)	16 (9.8)

a Table includes only intervention participants who did not refuse to be in the study and did not self-report as ineligible (n = 184).

b Chart review only excludes anyone for whom chart review data were unavailable (n = 21) or who was up-to-date with CRC screening at baseline based on the chart review (n = 21).

c Combined data excludes participants who were up-to-date with screening at baseline based on Medicaid claims data or chart reviews. Evidence of screening during the intervention is accepted from either source (chart reviews or Medicaid claims).

### Process outcomes

Among intervention participants who completed a survey (n = 56), most (51%) said that they were definitely interested in getting screened in the next 6 months and that they intended to ask for screening (60%). Colonoscopy, the preferred test, was selected by 35% of respondents; 27% preferred fecal occult blood test only, 10% preferred sigmoidoscopy alone or with fecal occult blood test, 25% were unsure, and 4% did not intend to get screened. Among survey respondents who answered the question about DVD ownership, 62% (30/48) reported having a DVD player at home. Among the 21 respondents who reported watching the DVD decision aid, most stated that the amount of information included was “about right” (13/21, 61.9%), that the information “was not upsetting” (66.6%), that the length was “just about right” (71.4%), that all of the information provided was clear (61.9%), that it was very helpful for making a decision (64%), and that they would recommend the decision aid (61.9%).

## Discussion

We found that our mailed DVD decision aid and telephone-based patient navigator intervention did not significantly increase CRC screening among Medicaid patients compared with matched controls. Our results differ from those of previous patient navigator interventions, which showed improvements in screening in vulnerable groups (9,10,16,17). Two studies used telephone-based counseling but included only those who agreed to participate in the final analysis (16,17). For our intent-to-treat analysis, we included all eligible participants so that we could estimate the effect of the intervention in the full eligible population, not just the subgroup that would agree to participate in a study.

In studies with similar methods, patient navigators reached 44.0% to 75.0% of patients compared with 26.7% in our study. Our navigator was unable to contact most patients over the telephone because of wrong or disconnected numbers and unanswered calls. Including in-person recruitment (9) and increasing the number and length of contacts (10) may have improved our reach. As was found in previous research, we found that screening was higher among participants who talked with the patient navigator (9,10). However, even those whom the navigator reached did not fully use the patient navigator’s services. Anecdotally, we found that many patients did not think they needed help or preferred to speak with their physician before making a decision.

Our study had some limitations. It was not randomized, but did use a matched control group. We controlled for demographic differences in our analysis to account for differential distribution of demographic characteristics, notably race, between groups. One limitation of using data from control practices whose patients were not enrolled in the study was that only claims data and not chart review data were available for the control group. Thus, we based the effectiveness analysis only on claims, which may have missed some screening, particularly through fecal occult blood testing, which is not always billed. We also included people who may have already been screened, which potentially led to underestimation of outcome estimates.

This study has several strengths. We targeted a vulnerable population with substantial comorbidities and socioeconomic barriers to screening. We also conducted the study in partnership with CCNC, which allowed us to better understand how similar programs could be integrated into the current health care infrastructure and possibly disseminated across the state.

Because contacting patients was difficult, future studies could increase intervention reach and use by basing the work in a clinical setting. Previous studies using decision aids and health educators playing a similar role to a navigator have been effectively delivered in a clinic setting (18,19). Moreover, most (84.3%) of patients in our study had at least 1 visit during the 6-month study period, suggesting potential opportunities to meet the navigator in the clinic, establish rapport, and project stronger physician endorsement of the intervention. Our results suggest that if reach and use were improved, the decision aid plus patient navigator intervention could increase CRC screening among vulnerable patients.
